# 

**Published:** 1914-02-21

**Authors:** 


### GIFTS TO ADDENBROOKE'S HOSPITAL.

Professor J. B. Bradbury, M.D., F.R.C.P., Senior Honorary Physician to Addenbrooke's Hospital, has given £21 to the hospital, which constitutes a Life Governorship; Mr. Charles Finch Foster, J.P., has given £100, constituting him also a Life Governor; Mr. Guggenheim, of Cambridge, has given £25, constituting a Life Governorship; and the Executors of the late W. B. Gurteen, of Haverhill, have handed over a legacy of £500, duty free. A cot in the hospital will be named after the late William Basham Gurteen, and Mr. Jabez Gurteen, of Coupals, Sturmer, Essex, senior executor, will be appointed an Honorary Governor.

The President, Colonel the Hon. Harry L. W. Lawson, M.P., has consented to occupy the chair at the seventy-fifth annual general meeting of the Newsvendors' Institution on Thursday evening, February 26, at seven o'clock, at Memorial Hall, Farringdon Street. Recommendations to increase the pension list and to amend the Society's rules will be discussed.

### BEQUESTS FOR MEDICAL MISSIONS.

Mr. Robert Hamlyn Hooker, of Weston-super-Mare, has bequeathed £1,000 to the Weston-super-Mare Hospital, and £1,000 to the Church Missionary Society for the maintenance of their Medical Hospital at Srinagar, Kashmir. Subject to one life interest, he left £500 to the Weston-super-Mare Hospital, and the ultimate residue of his property, subject to various legacies and annuities, he left to the Church Missionary Society, for the maintenance of its medical missions.

RATES OF SUBSCRIPTION: Twelve months, 6/6; Six months, 4/-; Three months, 2/-, post free to any address in the United Kingdom. Abroad, Twelve months, 8/8; Six months, 5/-; Three months, 2/6, post free.—Address The Subscription Dept., "THE HOSPITAL," The Hospital Building, 28/29 Southampton Street, Strand, London, W.C.

